# Characterization of culturable airborne bacteria and antibiotic susceptibility profiles of indoor and immediate-outdoor environments of a research institute in Ghana

**DOI:** 10.12688/aasopenres.12863.2

**Published:** 2018-08-20

**Authors:** Isawumi Abiola, Adiza Abass, Samuel Duodu, Lydia Mosi

**Affiliations:** 1 West African Centre for Cell Biology of Infectious Pathogens (WACCBIP) , University of Ghana, Accra, LG 54 , Ghana; 2 Department of Biochemistry, Cell and Molecular Biology, University of Ghana, Accra, LG 54, Ghana

**Keywords:** Airborne Bacteria, Antibiotic resistance, Indoor Air, Bacteriological profile

## Abstract

**Background:** The study was conducted to determine the bacterial composition and antibiotic susceptibility profiles of a research institute at the University of Ghana where workers and students spend about 70-85% of their lives in indoor and immediate-outdoor environments. This is imperative as one-third of the recognized infectious diseases are transmitted through airborne-route. Furthermore, the increasing rate of bacterial antimicrobial resistance associated with such environments poses serious public health challenges.

**Methods:** A total of 42 airborne samples were collected from eight major sites at the Department of Biochemistry, Cell and Molecular Biology (BCMB), using passive bacterial sampling techniques. Standard phenotypic microbiological procedures were used to characterize the isolates. Antibiotic susceptibility profiles were determined using standard disk diffusion method and guidelines of Clinical and Laboratory Standards Institute (CLSI).

**Results:** Four groups of bacterial isolates were identified from the total samples collected with Gram positive bacilli as the most common. All the isolates showed resistance to beta lactam and sulfonamide classes of antibiotics with full resistance (100%) to ampicillin and penicillin. In total, seven different anti-biotypes were observed with the highest susceptibility displayed towards tetracycline and gentamycin. Significantly, the various air sampling sites of the institute indicated the presence of bacteria with the majority showing multiple antibiotics resistance.

**Conclusions: **Although the recovery of bacteria from supposed sterile environments calls for attention, the observed low contamination rate as compared to the WHO standard suggests a minimum risk of exposure of students and workers to airborne microbial contamination.

## Introduction

Quality of air, especially in indoor environments where people spend 80–95% of their lives is of significant health importance ^1^. Microorganisms are ubiquitous; they normally inhabit indoor and outdoor environments. The inhaled air in the indoor environment is dominated by a number of microorganisms, with consequent effects on the health those indoors ^2^. Little is known about the diverse communities of bacteria shared by indoor environments such as houses, offices, laboratories, schools, hospitals, and other indoor environments where people work, relax or find solace ^3, 4^. The diversity of these microbes in the indoor environment is influenced by several factors such as water, temperature, moisturized surfaces or worktops, the rate of particle deposition, and other parameters like indoor pollutants, especially those generated by various human activities ^5, 6^.

Bioaerosols, mostly bacterial and fungal spores are actively living complex particles that have been associated with contamination of indoor air ^5, 7, 8^. The presence of these biological contaminants has been reported in the air of hospitals ^9^, but little is known about their impact on a typical research environment in Ghana ^10^. As dangerous as bioaerosols are by themselves, they also secrete toxins that are transmitted by the airborne route through the nasal airways making indoor environment a potential source of human pathogens ^11^. Microorganisms gain access to different indoor compartments of buildings through openings like doors, windows, blowing fan blades, air conditioners and the immediate-outdoor environments ^3, 12^. Immediate-outdoors areas, usual described as 0.9m to 3.5 away from the main building, includes foyers, such as the corridor, lobby or hallway ^13^. In addition the indoor air microbiomes originating from outdoor air-space drifts ^14^ have influencing factors which account for diverse microbial distribution. In a typical research environment of academic training and learning, a series of movements do occur from the outdoors through the immediate-outdoors to indoors ^2, 14^. This facilitates the movement of microorganisms, especially bacteria to different compartments of the building ^15^.

Several species of microorganisms have been isolated across indoor environments in previous studies ^12^. Although most of these microbes have been reported as opportunistic pathogens, they are not necessarily associated with severe infections ^7, 16^. However, they can pose significant challenges to immune-compromised individuals ^17, 18^. Sterile conditions, especially in biological laboratories control microbial growth ^19^. Interestingly, the microbes are able to survive using the air routes to other favourable environments within indoor environments ^15^. The laboratory hoods, although they are meant to provide a sterile environment for designated experiments, could serve as potential site for bacterial contaminations when sterility is compromised.

An increase in bacterial antimicrobial resistance and emergence of new strains associated with academic research environments is a serious public health challenge and has become increasingly important in recent years. In an environment where inter-personal and research activities are so diverse, bacteria resistance to antimicrobials is a possibility ^20– 22^. Studies of indoor air qualities and antibiotic susceptibility patterns of bacterial isolates present in most public institutes in developing countries have not been reported so far. This study was designed to characterize the bacterial composition and antibiotic susceptibility patterns of isolates recovered from indoor and immediate-outdoor air of a tertiary research institute in Ghana.

## Methods

### Sampling sites

**Figure 1.**
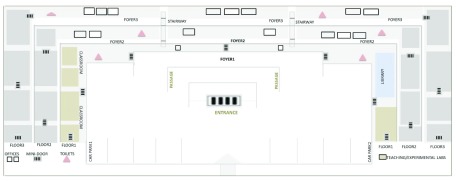
Graphical Diagram of Sampling Sites.

This study was conducted between January and May 2017. The study involved determination of bacterial loads and antibiotics susceptibility profiles of the air in selected study sites within the indoors and immediate-outdoors (foyers) of a research institute at the Department of Cell and Molecular Biology (BCMB). Sampling was conducted at different times within the day in duplicates for a period of eight weeks between temperature ranges of 20–32°C. Sampling sites included teaching laboratories (33 × 75 feet), classrooms (32 × 45 feet), experimental laboratories (28 × 40 feet and 12 × 22 feet), laboratory biosafety hoods, foyers (35 × 170 feet and 35 × 40), toilets (8 × 12 feet) and the library (36 × 41 feet) ( Figure 1).

### Cultivation of samples

All samples were collected using the open plate passive sampling technique and processed under aseptic conditions following standard microbiological methods ^5, 11, 23^. Non-sampled closed plates as controls were included alongside the experiment. Nutrient agar (Oxoid, England, CM0003), MacConkey agar (Oxoid, England, CM0007B), Blood agar (Oxoid, England, CM0055) and Mannitol salt agar (Oxoid, England, CM0085) plates were exposed for 60 minutes during daily active working hours (8am – 5pm) at different sites. The plates and non-sampled plates controls were incubated at 37°C under aerobic conditions for 24–48 hours.

### Isolation and identification of isolates

Bacteria isolates were identified using phenotypic microbiological methods described by Aguilera-Arreola *et al.* (2016) ^24^. Microscopy (Gram’s staining) and biochemical reactions were performed ^19, 25^. Standard plate count was performed to determine the bacterial loads across the sampled sites ^12^. The quantitative measurements of bacteria in colony forming unit per m ^3^ was determined using an equation adapted from Samuel Fakedu ^26^:

Colony forming unit (cfum ^-3^) = 5(Number of colonies per Petri dish) × 10 ^4^ / Dish surface (cm ^2^) × Exposure time (minutes)

### Frequency of outdoor-indoor movements

The frequency of movements from outdoor to indoor environments was determined for a period of one month using manual counting and closed circuit television camera monitoring system.

### Antimicrobial susceptibility testing

Clinical Laboratory Standards Institute (CLSI 12 ^th^ Edition) guidelines were followed to carry out the Antimicrobial susceptibility testing using disk diffusion method. Commonly used antibiotics prescribed by clinicians were selected, based on their general known effectiveness against bacterial infections. The discs used for screening Gram positive and negative bacteria contained the following antibiotics with the respective concentrations: ampicillin (10 μg), cefotaxime (30μg), chloramphenicol (30μg), ciprofloxacin (5 μg), gentamicin (10 μg), nalidixic acid (30 μg), nitrofuratoin (200μg), tetracycline (30μg), penicillin (15 μg), flucloxacillin (5 μg), cloxacillin (10 μg), erythromycyin (5 μg), ceftriaxone (30 μg) and cotrimoxazole (25 μg) (Mast Diagnostics, Mast Group Ltd., Merseyside, U.K.).

### Statistical analysis

All statistical analyses were performed using SPSS package version 17.0 and Graphad prism version 6 software. *P*-values less than 0.05 were considered statistically significant.

## Results

### Total isolated bacteria and diversity across sites

**Figure 2.**
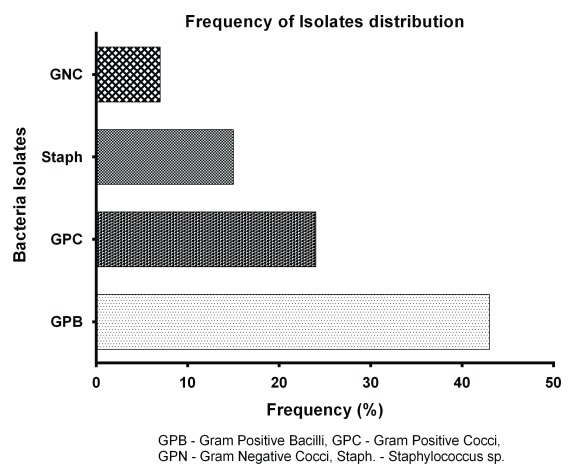
Distribution of bacterial isolates ( **GPB**: Gram positive bacilli, **GPC**: Gram positive cocci, **GNB**: Gram negative bacilli, Staph – *Staphylococcus* spp.).

Of the 42 total samples collected 87% of the isolates recovered were identified as Gram positive bacilli, *Staphylococcus* sp., Gram positive cocci and Gram negative bacilli, ( Table 1 and Figure 2).

**Table 1.** Phenotypic characteristics of isolates.

Biochemical Tests	Probable Bacteria sp.			
	GPB	GPC		GNB
		Staph. sp.	Strep. sp.	
Catalase	+	+	-	+/-
Motility	+	-	+	+/-
Starch Hydrolysis	+	-	+	-
Hemolysis	+	-	+	
Acid Production from Mannitol	+	+	-	+
Citrate	+	+	+/-	+
Nitrate reduction	+			
H _2_S	+	-	-	+/-
Urease		+		
Oxidase		-	+/-	
Coagulase	-	+		
Lipase		+		
Ornithine Decarboxylase		-		

**Figure 3.**
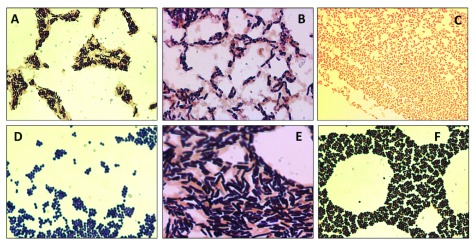
Microscopy Results of Representative Isolates. **A**, **E** – Gram positive bacilli, **B** – Gram positive bacilli with spores unable to pick the staining dye, **D**, **F** – Gram positive cocci, **C** – Gram negative short rods (a representation of three different replicates).

+: Positive, - : Negative ( **GPB**: Gram positive bacilli, **GPC**: Gram positive cocci, **GNB**: Gram negative bacilli, Staph – *Staphylococcus* spp., Strep - Streptococcus),

*Staphylococci* were isolated on Mannitol salt and nutrient agar media. *Staphylococcus aureus* was identified as mannitol fermenting colonies. Catalase positive isolates were classified as *Staphylococcus* species differentiating them from *Streptococcus* which are negative for catalase activities ( Table 1). Haemolysis was used to further confirm the *Streptococcus* species by checking their activities on Blood agar ( Table 1). The identification was further confirmed as positive cocci with Gram’s reaction and microscopic examination, signifying the trapping of the staining dye in the peptidoglycan layer of the organism cell wall ( Figure 3).

Gram positive bacilli were isolated from blood and nutrient agar media after an overnight growth ( Table 1). Microscopy further confirmed the identification as Gram positive bacilli, mostly in chains ( Figure 3). Gram negative bacilli were isolated from MacConkey agar medium after an overnight incubation period. The rose-pink colouration of the medium differentiates the lactose fermenters from the non-lactose fermenters ( Table 1). The identifications were confirmed with characteristic appearance as pink rods after Gram staining ( Figure 3).

### Diversity and predominant isolated bacteria across sites

**Figure 4.**
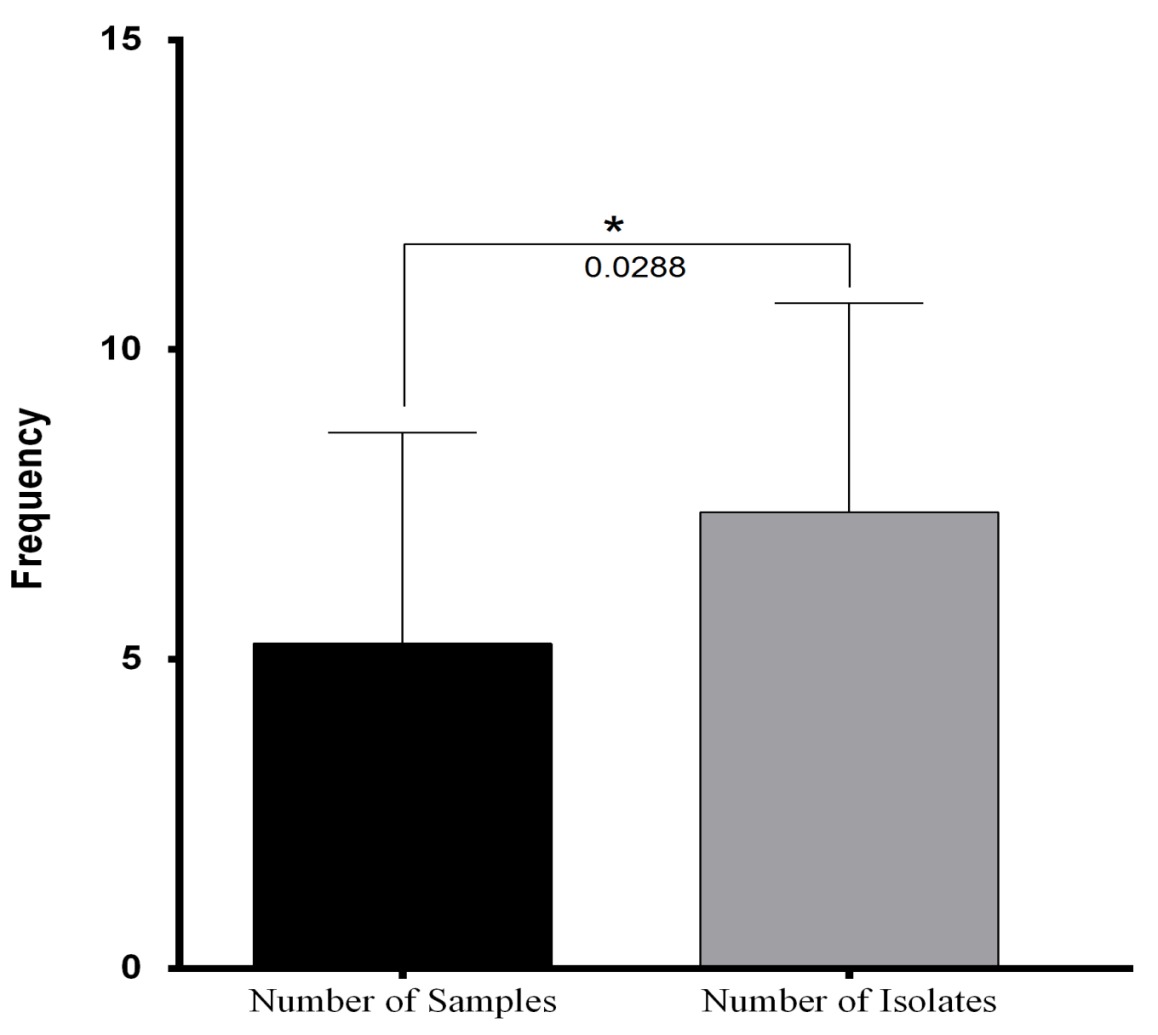
Significance (p < 0.01) of the bacterial isolates with respect to sampling across the site.

**Figure 5.**
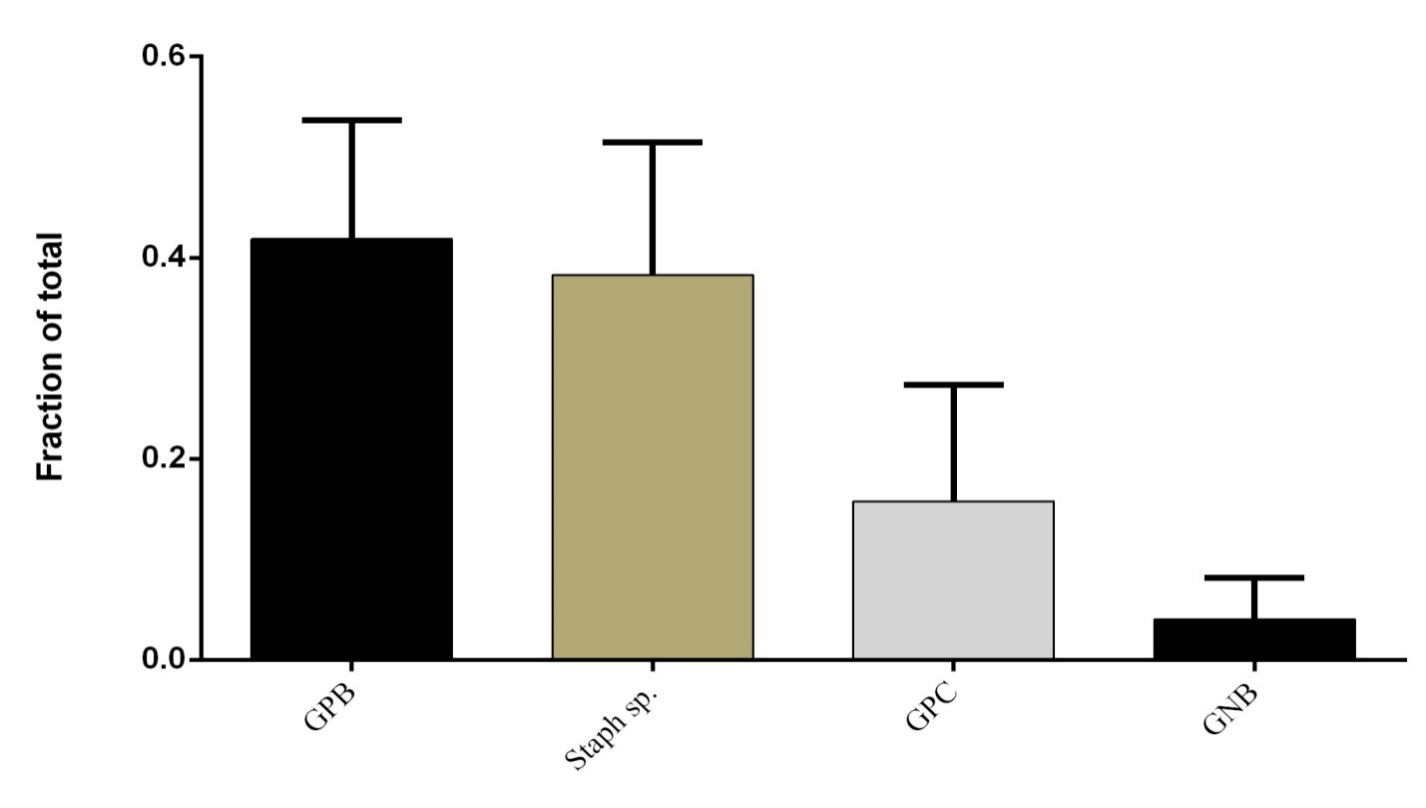
Most common bacteria appearance across the sampling sites ( **GPB**: Gram positive bacilli, **GPC**: Gram positive cocci, **GNB**: Gram negative bacilli, Staph – *Staphylococcus* spp.).

The percentage of bacteria diversity isolated across the different sites is presented in ( Table 2), with a total of 54 isolates belonging to three the genera identified. A moderate degree of significant correlation was observed between the number of samples and the isolates recovered. The highest number of isolates was obtained from the foyers ( *n*=13), followed by the toilets ( *n*=11), then the classrooms ( *n*=9) and finally from the library ( *n*=7). The lowest number of isolates was from the railings of the stairways ( *n*=2). A significant number of diverse bacteria was identified from the samples collected across the sampled sites ( Figure 4). The most commonly isolated bacteria across the sampling sites are Gram positive bacilli, with highest percentage in the foyers and toilets as compared to the classrooms and library ( Figure 5).

**Table 2.** Diversity of bacteria isolated across the sites with respect to size.

Sampling Sites	Number of Samples (per site)	Number of Isolates (per site)	Genera	Size (mmsq)
Railings	2	3	GPB, GPC	
Library	3	7	GPB, GPC, GNB	
Toilet	6	11	GPB, GPC, GNB	
Teaching Lab	4	5	GPB, GPC	
Classrooms	4	9	GPB, GPC, GNB	
Foyers	6	13	GPB, GPC, GNB	
Experimental Lab	13	6	GPB, GPC	
Lab Biosafety Hood	4	5	GPB, GPC	

**GPB**: Gram positive bacilli, **GPC**: Gram positive cocci, **GNB**: Gram negative bacilli, Staph – *Staphylococcus* sp., Strep - Streptococcus

To compare the average percentage of the bacterial composition of both indoor and immediate-outdoor air, the results were also reported as the number of colony forming unit (CFUm ^-3^) ( Table 3). The bacterial concentration is within the range 54 – 249 CFUm ^-3 ^with the foyers having the highest and the railings the lowest. In consideration of the total bacteria concentrations across the sites, indoors had the higher bacteria representation as compared to the outdoors. There is lower degree of correlation between the samples collected from the sites and the colony forming unit. Frequent movements of students and workers from immediate-outdoor to indoor environments were determined with a daily minimum of 210 and maximum of 315 people ( Table 4).

**Table 3.** Total number of bacteria in cfum ^-3^.

Sample Grade	Sampling Sites	Total number of bacteria (cfum ^-3^)
1	Railings	0.54×10 ^2^
2	Library	1.34×10 ^2^
3	Toilet	2.06×10 ^2^
4	Teaching Lab	1.21×10 ^2^
5	Classrooms	1.76×10 ^2^
6	Foyers	2.49×10 ^2^
7	Experimental Lab	1.02×10 ^2^
8	Lab Fume Hood	0.89×10 ^2^

**Table 4.** Frequency of Outdoor-Indoor Movements.

Day	Active Working Hours									
	manual count				Average	cctv camera count				Average
	wk1	wk2	wk3	wk4		wk1	wk2	wk3	wk4	
Day 1	201	186	243	211	**210**	321	207	243	401	**293**
Day 2	281	142	179	292	**224**	181	242	449	378	**315**
Day 3	181	253	129	307	**218**	281	253	329	307	**293**
Day 4	282	142	201	262	**221**	382	164	206	282	**259**
Day 5	196	263	187	289	**234**	196	286	281	248	**252**

wk –week, cctv – closed circuit television

### Antimicrobial susceptibility patterns

**Figure 6.**
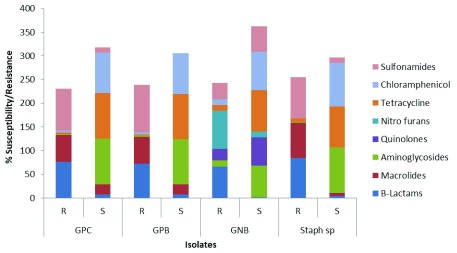
Percentage Susceptibility of the Isolates to Different Classes of Antibiotics ( **GPB**: Gram positive bacilli, **GPC**: Gram positive cocci, **GNB**: Gram negative bacilli, Staph – *Staphylococcus* spp., **R** – resistant, **S** – susceptible).

All the 54 isolates were tested against fourteen different selected antibiotic discs, belonging to eight different classes of antibiotics ( Table 5). Control strains of *E. coli* and *S. aureus* (obtained from Mosi Bacteriological Lab) with established antimicrobial profiles were included alongside the experiment. The antimicrobial resistance profile and susceptibility patterns showed that 87.7% and 76.7% of the Gram positive cocci were resistant to Beta Lactam and sulfonamides, 57.1%, 72.4% and 100% of Gram positive bacilli were resistant to Beta Lactam, macrolides and sulfonamides. Resistance to Nitrofurans by Gram negative bacilli was 80.4%, while 84.2% and 87.7% of *Staphylococcus* species showed resistance to sulfonamides and Beta Lactam respectively. Resistance to flucloxacillin across the isolates was observed; highest with *Staphylococcus* sp. Susceptibility of the isolates to tetracycline and gentamycin were observed especially with some Gram positive isolates ( Figure 6). All the isolates showed resistance to at least 2-classes of the 8 different classes of antibiotics tested. Seven different anti-biotypes (multiple antibiotic resistance patterns) were observed across the isolates with a minimum of resistance to two different antibiotics and maximum of nine different antibiotics ( Table 6).

**Table 5.** Percentage Frequency of Isolates to Antibiotics.

Antibiotics Tested	Disc Potency (ug)	Frequency (%)							
		GPC		GPB		GNB		Staph. spp.	
		S	R	S	R	S	R	S	R
**Flucloxacillin**	5	0	85.7	11.2	80.0	10.5	71.3	0	88.7
**Erythromycin**	5	20.7	57.1	0	83.3	NT	NT	5.5	74.3
**Cloxacillin**	5	3.6	64.3	0	50.0	NT	NT	3.6	64.3
**Ceftriaxone**	30	15.3	58.6	12.5	68.3	2.7	95.0	15.3	58.6
**Cotrimoxazole**	25	11.2	87.7	0	100.0	53.4	34.4	11.2	87.7
**Nitrofuratoin**	200	NT	NT	NT	NT	11.5	80.4	NT	NT
**Chloramphenicol**	30	85.7	4.2	66.6	6.7	81.4	11.6	92.6	0
**Tetracycline**	10	96.0	2.5	97.0	0	87.6	12.1	85.7	7.1
**Cefotaxime**	10	12.1	57.1	9.5	67.0	NT	NT	13.8	77.6
**Cefuroxime**	30	22.6	71.4	21.1	41.6	0	100.0	0	100.0
**Penicillin**	15	0	100.0	0	100.0	0	100.0	0	100.0
**Ampicillin**	10	0	100.0	0	100.0	0	100.0	0	100.0
**Nalidixic Acid**	30	NT	NT	NT	NT	60.0	24.3	NT	NT
**Gentamicin**	10	97.0	2.0	95.5	2.6	66.7	13.2	97.0	2.0

**R** – Resistance, **S** – Susceptible, NT – Not Tested (Antibiotics were not available at the time of this experiment) **GPB**: Gram positive bacilli, **GPC**: Gram positive cocci, **GNB**: Gram negative bacilli

**Table 6.** Multiple Antibiotic Resistance Patterns of the bacterial Isolates.

Isolates	Level	Type of Antibiotics	Antibiotic Classes	N ^o^ Isolates
**Gram Positive Cocci**	Max. (9)	FLX, ERY, CX, CTX, COT, CFX, CXM, PEN, AMP	B-Lac, Mac, Sun	11
	Min. (3)	C, TET, GEN	CH, TE, AMIN	6
**Gram Positive Bacilli**	Max. (8)	FLX, ERY, CX, COT, CFX, PEN, AMP, CRX	B-Lac, Mac, Sun	7
	Min. (2)	GEN, C	AMIN, CH	3
**Gram Negative Bacilli**	Max. (8)	FLX, CFX, NIT, CTX, PEN, AMP, NAL, COT	B-Lac, Nitro, Qui, Sun	3
	Min. (3)	C, TET, GEN	CH, TE, AMIN	3
***Staph* sp.**	Min. (9)	FLX, ERY, CX, CFX, COT, CXM, CTX, PEN, AMP	B-Lac, Mac, Sun	9
	Max. (2)	TET, GEN	TE, AMIN	5

FLX – flucloxacillin, ERY – erythromycin, CX – cloxacillin, CTX – ceftriazone, NIT – nitrofuratoin, PEN – penicillin, AMP – ampicillin, NAL – nalidixic-acid, C – chloramphenicol, TET – tetracycline, GEN – gentamycin, COT – cotrimoxazole, CRX – cefuroxime, CFX - cefotaxime

## Discussion

The study considered bacterial isolates in the indoor and immediate-outdoor air environments of a research institute in Ghana. It was observed that all the sections sampled showed diverse bacterial loads similar to other studies conducted elsewhere ^7, 15^. In accordance with this study, frequent movements of students and workers from immediate-outdoor to the indoor environments decisively influenced the diversity and abundance of the isolated bacteria. In this context, samples collected from different sections were significantly matched to the bacteria isolated.

The inflow of air through the immediate-outdoors and other openings, like the doors which are been engaged daily and almost every minute by the students and workers alike contributed to the high frequency of bacteria obtained in this study. This data supports a study conducted on understanding airborne microbial dynamics in built environments, which indicated that indoor airborne bacterial communities are influenced by outdoor air source and ventilation ^27^. Classrooms and laboratories sampled were air-conditioned; therefore bacterial contamination of air reported in this study is inevitable, especially when the air conditioner blades are not properly or frequently cleaned. Similar results and observation have been reported ^7^, which emphasized blowing-air blades as potential microbial sources ^3, 11^. The foyers, regarded as an immediate-outdoor environment had a low bacteria representation as compared to the indoor environment. Although outdoor air has been reported as a major driver of the indoor air microbiome ^3^, our data suggests higher bacterial concentrations in the indoor environments. It could be that in addition to human occupancy and activities, the outdoor-indoor bacterial penetration were effective, thereby contributing to the high indoor bacterial loads ^11^.

The toilet is a small area of the building but visited by almost all the students and workers. Small areas with a lot of people have been reported to influence the concentration of bacteria ^20, 23, 28, 29^. The high percentage of bacteria in toilets could be associated with lack of proper disinfection practice, low level of hygiene, and shedding of human microflora ^3, 29^, with high potentials to be propagated into the air wave. The library and classrooms had a higher percentage of contamination with indoor bacteria when compared to teaching and experimental laboratories ^2, 30^. The results are similar to a study conducted on the assessment of bacteria in indoor air of a medical college ^19^. It is also interesting to mention that the experimental laboratories were more contaminated than the teaching laboratories. This may be due to diverse research activities in the experimental laboratories which suggest a need for more cautionary measures in basic routine laboratory operations. A study conducted on the analysis of variation in total airborne bacteria concentration in microbial laboratories reported improper disinfection practice and handling of specimens without following the basic rules of sterility as a possible contributing factor ^13, 31^. Moreover, the population of students in the classrooms and library is also a possible contributing factor to the higher bacteria concentrations in these environments.

As expected, the laboratory biosafety hood had a relatively low percentage of bacterial concentration. Isolation of bacteria from the hoods appears inappropriate as the UV light shield and creates sterile conditions. However, the reasons for the presence of bacteria, albeit at low numbers, might be due to improper disinfection practice, dilution factors of the disinfectants used or/and cross-contamination. In the study, the staircase railings had the lowest percentage of bacteria isolates, contrary to studies conducted elsewhere ^20^. Although the reason for this is clearly unknown, it could be attributed to the low samples collected ( *n*=2).

The resistance of the bacterial isolates to most of the antibiotics tested in this study calls for serious attention. Both Gram positive and Gram negative bacteria had higher rates of resistance to different classes of antibiotics. Most of the antibiotic classes were used as treatment options by clinicians in case of an infection in the study area. This might limit the antibiotic choice for the treatment of infections associated with these bacteria in the study area. Interestingly, gentamicin and tetracycline showed a level of effectiveness especially against some of the Gram positive bacteria, and these antibiotics might be considered as parts of the treatment regimen in the study area.

In conclusion, the various air sampling sites of the institute showed the presence of bacteria, though with low levels of contamination within the range (54 – 249 CFU/m ^3^) as compared to the World Health Organization standard. Thus, students and workers are at low risk of exposure to airborne bacteria. Isolation of bacteria from the laboratory biosafety hood is of great health concern. Although the majority seems opportunistic, they may have pathogenic potentials with significant consequences. The strength of this study is that it unravels the level of bacterial contamination and subsequent antibiotic susceptibility profiles of a typical working and learning research environments. The antibiotic profiles of the bacterial isolates from the study centre have not been conducted before; the data presented only suggest possible exposure to resistant bacteria strains.

Overall, proper disinfection practice, working under a standard sterile condition, quality monitoring of air and maintenance of devices that can transmit bioaerosol across different locations are highly recommended; for this will safeguard the health of students, staff, and workers.

## Data availability

The data underlying this study is presented in the tables with additional data available from Figshare. Dataset 1: S1_Isawumi Abiola *et al.* 2018.pdf. https://doi.org/10.6084/m9.figshare.6241829.v1 ^32^

This dataset is available under a CC BY 4.0 license

## References

[1] Dacarro C Picco AM Grisoli P: Determination of aerial microbiological contamination in scholastic sports environments. *J Appl Microbiol.* 2003;95(5):904–912. 10.1046/j.1365-2672.2003.02044.x 14633018

[2] Weikl F Tischer C Probst AJ: Fungal and Bacterial Communities in Indoor Dust Follow Different Environmental Determinants. *PLoS One.* 2016;11(4):1–10, e0154131. 10.1371/journal.pone.0154131 27100967 PMC4839684

[3] Hwang SH Park HH Yoon CS: Analysis of variation in total airborne bacteria concentration to assess the performance of biological safety cabinets in microbial laboratories. *Saf Health Work.* 2014;5(1):23–26. 10.1016/j.shaw.2014.01.001 24932416 PMC4048000

[4] Eames I Tang JW Li Y: Airborne transmission of disease in hospitals. *J R Soc Interface.* 2009;6 Suppl 6:S697–702. 10.1098/rsif.2009.0407.focus 19828499 PMC2843953

[5] Brodie EL DeSantis TZ Parker JP: Urban aerosols harbor diverse and dynamic bacterial populations. *Proc Natl Acad Sci U S A.* 2007;104:299–304. 10.1073/pnas.0608255104 17182744 PMC1713168

[6] Dellinger RP Levy MM Carlet JM: Surviving Sepsis Campaign: international guidelines for management of severe sepsis and septic shock: 2008. *Crit Care Med.* 2008;36(1):296–327. 10.1097/01.CCM.0000298158.12101.41 18158437

[7] Of J: Review Article.2014;5:7–12.

[8] Douwes J Thorne P Pearce N: Bioaerosol health effects and exposure assessment: progress and prospects. *Ann Occup Hyg.* 2003;47(3):187–200. 10.1093/annhyg/meg032 12639832

[9] Beggs CB: The Airborne Transmission of Infection in Hospital Buildings: Fact or Fiction? *Indoor and Built.* 2003;9–18. 10.1177/1420326X03012001002

[10] Elizabeth M: A Review of Nosocomial Infections in Sub-Saharan Africa. 2016;15(1):1–11. 10.9734/BMRJ/2016/25895

[11] Clau ßM: Particle size distribution of airborne micro- organisms in the environment - a review. 2015 10.3220/LBF1444216736000

[12] Sheik GB Ali Abd Al Rheam Al Al Shehri ZS: Assessment of Bacteria and Fungi in air from College of Applied Medical Sciences (Male) at AD-Dawadmi, Saudi Arabia. *Int Res J Biological Sci.* 2015;4(9):48–53. Reference Source

[13] Act B. *Building Regulations.* 2016;16.

[14] Tagoe DN Baidoo SE Dadzie I: Potential sources of transmission of hospital acquired infections in the volta regional hospital in Ghana. *Ghana Med J.* 2011;45(1):22–26. 10.4314/gmj.v45i1.68918 21572821 PMC3090097

[15] Fang ZC Gong Z Ouyang P: Characteristic and Concentration Distribution of Culturable Airborne Bacteria in Residential Environments in Beijing, China. 2014;14(3):943–953. 10.4209/aaqr.2013.04.0109

[16] Of GA: case study.2013;6391:212–222.

[17] Coelho AC García Díez J: Biological Risks and Laboratory-Acquired Infections: A Reality That Cannot be Ignored in Health Biotechnology. *Front Bioeng Biotechnol.* 2015;3:1–10, 56. 10.3389/fbioe.2015.00056 25973418 PMC4412124

[18] Prussin AJ 2nd Marr LC: Sources of airborne microorganisms in the built environment. *Microbiome.* 2015;3: 1–10,78. 10.1186/s40168-015-0144-z 26694197 PMC4688924

[19] Rahkonen P Ettala M Salkinoja-Salonen M: Airborne Microbes and Endotoxins in the Work Environment of Two Sanitary Landfills in Finland Airborne Microbes and Endotoxins in the Work Environment of Two Sanitary Landfills in Finland.2017;6826.

[20] Joseph A, Robert Wood Johnson Foundation: The Impact of the Environment on Infections in Healthcare Facilities.2006 Reference Source

[21] Townsend DE Naqui A: Comparison of SimPlate Total Plate Count test with plate count agar method for detection and quantitation of bacteria in food. *J AOAC Int.* 1998;81(3):563–9. 9606922

[22] Yadav J Kumar A Mahor P: Distribution of airborne microbes and antibiotic susceptibility pattern of bacteria during Gwalior trade fair, Central India. *J Formos Med Assoc.* 2015;114(7):639–646. 10.1016/j.jfma.2013.04.006 23742901

[23] Castellanos-are AP Camarena-pozos DA Castellanos-are DC: Indoor and Built Microbial contamination in the indoor environment of tanneries. 2016;25:524–540.

[24] Aguilera-Arreola MG: Identification and Typing Methods for the Study of Bacterial Infections: a Brief Review and Mycobacterial as Case of Study. *Arch Clin Microbiol.* 2016;7:1 Reference Source

[25] Filipiak M: Microbiological Quality of Indoor Air in University Rooms. 2007;16:623–632. Reference Source

[26] Fakedu S Getachewu B: Microbiological Assessment of Indoor Air of Teaching Hospital Wards: A case of Jimma University Specialized Hospital. *Ethiop J Health Sci.* 2015;25(2):117–22. 10.4314/ejhs.v25i2.3 26124618 PMC4478262

[27] Maedow JF Altrichter AE Kembel SW: Indoor airborne bacterial communities are influenced by ventilation, occupancy, and outdoor air source. *Indoor Air.* 2014;24(1):41–8. 10.1111/ina.12047 23621155 PMC4285785

[28] Li J Zhou L Zhang X: Bioaerosol emissions and detection of airborne antibiotic resistance genes from a wastewater treatment plant. *Atmos Environ.* 2016;121(Part B):404–412. 10.1016/j.atmosenv.2015.06.030

[29] Mentese S Tasdibi D: Airborne bacteria levels in indoor urban environments: The influence of season and prevalence of sick building syndrome (SBS). *Indoor Built Environ.* 2016;25(3):563–580. 10.1177/1420326X14562454

[30] Yassin MF Almouqatea S: Assessment of airborne bacteria and fungi in an indoor and outdoor environment. *Int J Environ Sci Tec.* 2010;7(3):535–544. 10.1007/BF03326162

[31] Hall GS Mangel JI: Rapid Biochemical Tests for the identification of Anaerobes. *American Society of Microbiology.* 2016 10.1128/9781555818814.ch4.9

[32] Isawumi A Abass A Duodu S: S1_Isawumi Abiola *et al* 2018.pdf.2018 10.6084/m9.figshare.6241829.v1

