# Supplementary material for: Characterization of culturable airborne bacteria and antibiotic susceptibility profiles of indoor and immediate-outdoor environments of a research institute in Ghana
Source: AAS Open Res. 2018 Aug 20;1:17. Originally published 2018 May 17. [Version 2] doi: 10.12688/aasopenres.12863.2 (PMC7118738; doi:10.12688/aasopenres.12863.2)
Supplement: Peer review history

# Peer review: Reviewer response for version 2

**DOI:** 10.21956/aasopenres.13976.r26580

**Published:** 2018-10-02

**Authors:** Pramod Kumar

**Affiliations:** 1 All India Institute of Medical Sciences, New Delhi , India

The manuscript can be accepted for indexing.

I confirm that I have read this submission and believe that I have an appropriate level of expertise to confirm that it is of an acceptable scientific standard.

# Peer review: Reviewer response for version 2

**DOI:** 10.21956/aasopenres.13976.r26579

**Published:** 2018-09-03

**Authors:** Daniel Nii Aryee Tagoe

**Affiliations:** 1 Department of Biology, Boston College, Newton, MA, USA

No further comments to make.

I confirm that I have read this submission and believe that I have an appropriate level of expertise to confirm that it is of an acceptable scientific standard.

# Peer review: Reviewer response for version 1

**DOI:** 10.21956/aasopenres.13929.r26499

**Published:** 2018-07-13

**Authors:** Daniel Nii Aryee Tagoe

**Affiliations:** 1 Department of Biology, Boston College, Newton, MA, USA

Suggested title: Characterization of culturable airborne bacteria and antibiotic susceptibility profiles of indoor and immediate-outdoor environments of a research institute in Ghana.

 Introduction: this is well written and covered all the different aspects such as spaces, contamination, environment, antibiotics and antibiotic resistance.

 Methods: 

 Sampling sites: This require more information and possibly pictures. This is because they affect sources, distribution and numbers of bacterial that will be sampled. Sizes of foyer and lecture theatres would have helped as well. 

 Control: Authors indicated incubating plates up to 48 h. Incubating a non-sampled plate for similar period will be a good control.

 Data:

 Authors could perform correlational analysis of location against cfu. Additionally, a correlation of frequency of sampling sites against cfu would also have strengthened the data.

 Conclusion:

 The above suggestions will greatly improve the data and strengthened the discussion.

I confirm that I have read this submission and believe that I have an appropriate level of expertise to confirm that it is of an acceptable scientific standard, however I have significant reservations, as outlined above.

# Response

**Published:** 2018-07-16

**Authors:** Abiola Isawumi

**Affiliations:** West African Centre for Cell Biology of Infectious Pathogens, Ghana

Excellent review and comments.

Title: The suggestion to state the specific location where the study was carried out is appreciated.

Sampling sites: Yes, it is possible to include the sizes of sampled-sites (foyers, classrooms, teaching and experimental laboratories and the library). For the pictures of the sampled-sites, this will be subjected to further consideration.

Controls: Sorry I failed to mention that unsampled controls were included. The controls are unopened media plates. They were subjected to the same conditions as the opened plates.

Data: We will consider the kind of information that would be provided by the correlation analysis. And this would be adjusted accordingly if it suits the purpose of the study.

# Peer review: Reviewer response for version 1

**DOI:** 10.21956/aasopenres.13929.r26429

**Published:** 2018-06-11

**Authors:** Pramod Kumar

**Affiliations:** 1 All India Institute of Medical Sciences, New Delhi , India

**Title:** Characterization of culturable airborne bacteria and antibiotic susceptibility profiles of indoor and immediate-outdoor environments of a research institute

 It need to be improved as (mention city and country in the title) -

 "Characterization of culturable airborne bacteria of indoor and immediate-outdoor environments of a research institute, Accra, Ghana".

**Material methods:**

 Mention detailed of sampling sites including a graphical diagram. Describe basis of number of samples collected from each site. The manuscript is very basic and need significant enrichment.  Mention climatic condition or meteorological factors during the sampling period.

 Improve methodologies for bacterial identification with special emphasis on identification of *Staphylococcus aureus. *All the mannitol fermenters on MSA agar plates are not *S. aureus *as some of the other Staph sps also ferment mannitol. Authors need to identify *S. aureus *by using coagulase and thermonuclease tests or by PCR. Authors may refer a paper entitled “Prevalence of Methicillin Resistant Staphylococcal Bioaerosols in and around Residential Houses in an Urban Area in Central India” (Journal of Pathogen doi.org/10.1155/2016/7163615) for identification of airborne *S. aureus*. Mention formula for quantitative measurement of bacteria (cfu/m ^3^).

**Results:**

 Major comments:

- Serious concern is lack of quality control strains antimicrobial susceptibility testing (AST). Authors should revise AST using *E. coli* ATCC25922 and *S. aureus* ATCC25923) as quality control strains.
- It would be better if authors mention statistical confidence/significance to represent comparative bacterial count in different sites.
- Authors are also advised to do statistical correlation analyses of outdoor-indoor movements with bacterial counts to represent effect of movements on indoor bacterial prevalence.

Minor comments: CFU is “colony forming unit” not “coliform forming unit”.

**Discussion**: needs more focus.

I confirm that I have read this submission and believe that I have an appropriate level of expertise to confirm that it is of an acceptable scientific standard, however I have significant reservations, as outlined above.

# Response

**Published:** 2018-08-21

**Authors:** Abiola Isawumi

**Affiliations:** West African Centre for Cell Biology of Infectious Pathogens, Ghana

Excellent review and comments.

All the suggestions suitable for the purpose of this study have been considered for article improvements.

Many thanks.
